# Emerging Trends in Neoadjuvant Chemotherapy for Ovarian Cancer

**DOI:** 10.3390/cancers13040626

**Published:** 2021-02-05

**Authors:** Ami Patel, Puja Iyer, Shinya Matsuzaki, Koji Matsuo, Anil K. Sood, Nicole D. Fleming

**Affiliations:** 1 Department of Gynecologic Oncology and Reproductive Medicine, The University of Texas MD Anderson Cancer Center, Houston, TX 77030, USA; abpatel2@mdanderson.org (A.P.); puja.v.iyer@gmail.com (P.I.); asood@mdanderson.org (A.K.S.); 2 Department of Obstetrics and Gynecology, Osaka University Graduate School of Medicine, Osaka 565-0871, Japan; zacky@gyne.med.osaka-u.ac.jp (S.M.); koji.matsuo@med.usc.edu (K.M.); 3 Norris Comprehensive Cancer Center, University of Southern California, Los Angeles, CA 90033, USA

**Keywords:** neoadjuvant chemotherapy, epithelial ovarian cancer, targeted therapy, personalized treatment, optimal cytoreduction, laparoscopy scoring, molecular markers, tumor-based genetic markers, radiology-based models, interval tumor reductive surgery

## Abstract

**Simple Summary:**

Epithelial ovarian cancer is one of the most lethal cancers in women and is typically diagnosed at an advanced-stage. Historically, primary tumor reductive surgery was attempted followed by postoperative chemotherapy in most patients diagnosed with advanced ovarian cancer. However, neoadjuvant chemotherapy followed by interval tumor reductive surgery is an alternative approach for patients with advanced-stage ovarian cancer where primary tumor reductive surgery is not feasible. Here, we review proposed models that can assist in selecting patients who would benefit most from neoadjuvant chemotherapy followed by surgery.

**Abstract:**

Epithelial ovarian cancer remains a leading cause of death amongst all gynecologic cancers despite advances in surgical and medical therapy. Historically, patients with ovarian cancer underwent primary tumor reductive surgery followed by postoperative chemotherapy; however, neoadjuvant chemotherapy followed by interval tumor reductive surgery has gradually become an alternative approach for patients with advanced-stage ovarian cancer for whom primary tumor reductive surgery is not feasible. Decision-making about the use of these approaches has not been uniform. Hence, it is essential to identify patients who can benefit most from neoadjuvant chemotherapy followed by interval tumor reductive surgery. Several prospective and retrospective studies have proposed potential models to guide upfront decision-making for patients with advanced ovarian cancer. In this review, we summarize important decision-making models that can improve patient selection for personalized treatment. Models based on clinical factors (clinical parameters, radiology studies and laparoscopy scoring) and molecular markers (circulating and tumor-based) are useful, but laparoscopic staging is among the most informative diagnostic methods for upfront decision-making in patients medically fit for surgery. Further research is needed to explore more reliable models to determine personalized treatment for advanced epithelial ovarian cancer.

## 1. Introduction

**Figure 1:**
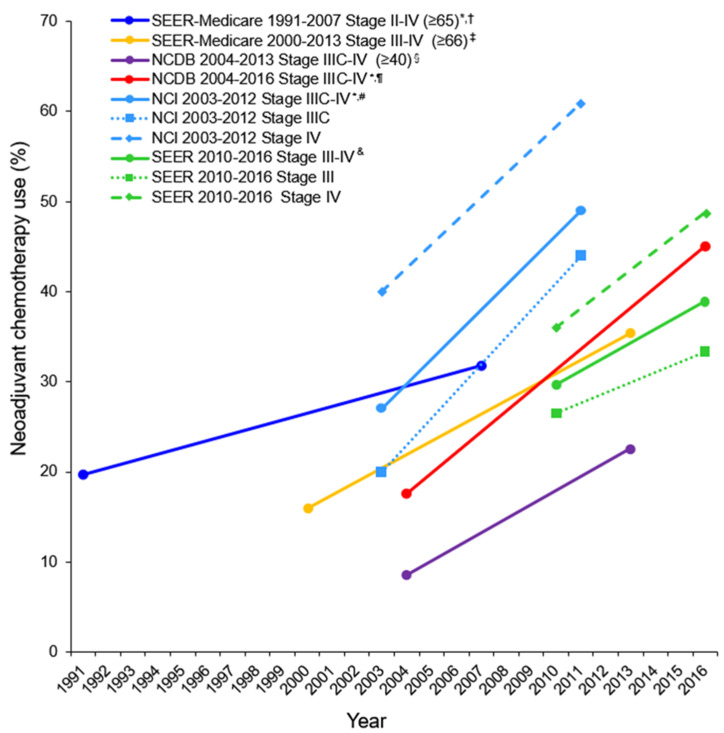
Trends of neoadjuvant chemotherapy use for advanced ovarian cancer in major U.S. observational studies. Adopted and modified from the author’s own work [11]: *Gynecol. Oncol.* **2021**, *160*, 32–39. Matsuo, K., et al. Possible candidate population for neoadjuvant chemotherapy in women with advanced ovarian cancer. Trends in the use of neoadjuvant chemotherapy for advanced ovarian cancer in U.S. observational studies. Copyright (2021) with permission from Elsevier. Proportion of NACT with ovarian cancer per calendar year is shown. Only starting point and end point are shown. Y-axis is truncated to 0–70%. * In the trend analysis, neoadjuvant chemotherapy without surgery was included ^†^ [8]. ^‡^ [9]. ^§^ [6]. ^¶^ [7]. ^#^ [10]. ^&^ [11]. Abbreviations: NCDB, National Cancer Database; and SEER, The Surveillance, Epidemiology and End Results; NCI, National Cancer Institute; U.S., United States of America.

Epithelial ovarian cancer remains the fifth leading cause of death among women in the United States, with an incidence of 10.4/100,000 women/year and a death rate of 6.9/100,000 women/year in the 2017 statistics report [1]. The American Cancer Society predicted about 21,750 new cases and about 13,940 deaths among U.S. women due to epithelial ovarian cancer in 2020 [2]. Approximately 52% of epithelial ovarian cancers are high-grade serous ovarian carcinoma (HGSOC) and are diagnosed at an advanced stage III (51%) or IV (29%) [3]. Ovarian cancer is typically diagnosed at a later stage primarily because of the presence of non-specific symptoms and a lack of effective screening methods; the 5-year relative survival rate is 48.6% based on the 2010–2016 statistics [1,4,5]. For years, primary tumor reductive surgery (pTRS) followed by platinum-based adjuvant chemotherapy has been the standard treatment for women with advanced disease. However, neoadjuvant chemotherapy (NACT) has gradually become an accepted alternative for certain patients for first-line treatment. The rate of NACT use has increased from 8.6% to 22.6% between 2004 and 2013 [6] and from 17.6% to 45.1% between 2006 and 2016 for advanced ovarian cancer [7]. The use of NACT has increased annually by 10.3% between 2011 and 2016 compared with an annual increase of 7.9% between 2006 and 2011 [7]. The results of NACT trends in several major U.S. observational studies are displayed in Figure 1 [6,7,8,9,10,11]. Overall, the NACT use has increased significantly in recent years particularly for patients with stage IV disease. The increase in the NACT use is also occurring in areas other than United States; for instance, the nationwide utilization of NACT has doubled from 2002–2015 in Japan [12].

Platinum-based drugs with paclitaxel are part of standard NACT regimen that have been used for newly diagnosed advanced ovarian cancers; however, limited information is available on use of bevacizumab (anti-angiogenesis drug), poly (ADP-ribose) polymerase (PARP) inhibitors and immunotherapy in NACT setting. Combination of bevacizumab with standard regimen in NACT setting demonstrated improved PFS among patients with advanced ovarian cancer [13,14]. Few phase III clinical trials are being conducted to determine the efficacy of combination of immunotherapy and platinum-based chemotherapy among patients with advanced ovarian cancer; results are pending [15,16].

The purpose of NACT is to decrease the tumor load and to increase the likelihood of achieving optimal cytoreduction (no visible residual disease (RD) or RD ≤ 1 cm) at interval tumor reductive surgery (iTRS). Various diagnostic models have been developed to determine the ideal upfront treatment strategy for patients with advanced-stage ovarian cancer with no clear “winner.” In this review, we provide a critical discussion of the available data and evolving models to aid in decision-making and guide future research.

## 2. Survival Effects of NACT

### 2.1. Clinical Trials Evaluating NACT

A summary of the key studies is displayed in Table 1. The results of two phase III randomized controlled trials (CHORUS and EORTC-55971) showed that NACT followed by iTRS has equivalent benefits on progression-free survival (PFS) and overall survival (OS), better optimal cytoreduction rates and low occurrence of perioperative morbidity and mortality among women (EORTC-55971) with advanced-stage ovarian cancer compared with results observed with pTRS followed by adjuvant chemotherapy [17,18]. A recent pooled analysis of these two trials showed that with long-term follow-up of nearly 8 years NACT and pTRS had comparable OS (hazard ratio 0.97, 95% confidence interval 0.86–1.09, *p* = 0.586) [19].

The Japan Clinical Oncology Group study (JCOG-0602) [22] and the SCORPION trial [21] reported shorter duration of surgery, shorter total surgery time, smaller amounts of blood loss during surgery and less frequent need for transfusion during treatment in patients who were treated with NACT followed by iTRS than in patients who underwent pTRS. In addition, the Japan Clinical Oncology Group study (JCOG-0602) study [22] observed less frequent abdominal organ resection and/or distant metastases, fewer deaths within 28 days of surgery and less invasiveness of iTRS in the NACT group.

A potential criticism of both the CHORUS and EORTC-55971 trials is the low optimal cytoreduction rates and the selection of patients with higher tumor burden [23]. A meta-analysis of four randomized trials [12,24] reported similar benefits of OS and PFS; a higher rate of optimal cytoreduction; a lower rate of serious adverse events related to surgery; a lower rate of grade 3 or 4 postoperative complications including gastrointestinal fistula, infection and death within 28 days; better quality of life; and less than 1% postsurgical mortality in patients treated with NACT followed by iTRS compared with findings in patients who underwent pTRS followed by adjuvant chemotherapy. Mueller and colleagues [25] observed higher optimal resection rates (80–81%), OS (72 months) and PFS (21 months) among patients treated with pTRS. The differences in results were thought to be primarily due to the selection of older patients and patients with higher tumor burden in the NACT group [25].

Optimal endpoints for demonstrating effectiveness of NACT trials are not yet fully known. The fifth ovarian cancer consensus conference of the gynecologic cancer intergroup summarized that OS and PFS are commonly used primary endpoints for first line clinical trials. Quality of life assessment, total gross resection rates, pathologic complete response rates, treatment response rates and molecularly defined response rates can be used as endpoints for NACT studies [26]. The FDA ovarian cancer clinical trial endpoints workshop mentioned that although OS and PFS are widely used primary endpoints, routine use of these endpoints for NACT trials is debatable. Pathologic complete response rates can be used as alternate endpoint for NACT trials. In addition, biomarkers such as cancer antigen 125, circulating tumors cells, circulating cell-free tumor DNA and exosomes may be used as translational endpoints in clinical trials [27].

### 2.2. Observational Studies Evaluating NACT

Summary of major U.S. observational studies is shown in Table 2. When it comes to “real-world practice”, the results are heterogeneous across the study populations [6,7,8,9,10,11]. For instance, results from a multicenter retrospective study among the National Cancer Institute designated centers in the United States noted better clinical outcomes in patients treated with pTRS, but higher complete cytoreduction rates in patients treated with NACT. The best median OS was 106 months in patients with optimal cytoreduction after pTRS and the worst median OS was 36 months in patients with RD after NACT [28]. Meyer and colleagues [10] noted a significant decrease in OS among patients with stage IIIC ovarian cancer who had been treated with NACT compared with those who had undergone pTRS. Patients with stage IV cancer in both groups showed no significant difference in OS that the results are opposite from a pooled analysis of two European trials [19]. Adjusted analyses of the data demonstrated decreased rates of intensive care unit admission or re-hospitalization among patients treated with NACT. They also found that NACT was associated with lower likelihood of ≥1 cm gross RD after iTRS [10]. Most of these U.S. observational studies found that NACT is associated with decreased survival versus pTRS (Table 2).

Survival effects of NACT may depend on patient and tumor factors, as shown in a recent analysis of U.S. tumor registry [11]. Among older patients, those with stage IV disease or those with higher extent of disease, NACT and pTRS had comparable survival. Moreover, NACT was associated with decreased mortality in old women and those with stage IV disease. In contrast, pTRS was associated with superior survival compared to NACT among younger patients, those with stage III disease and those with lesser extent of disease [11].

A meta-analysis of 17 published studies reported that patients who underwent pTRS had higher morbidity and mortality rates. Patients who had been treated with NACT followed by iTRS had shorter surgery time, less blood loss during surgery and shorter hospital admission stay. NACT was associated with a significant increase in complete/optimal cytoreduction rates; however, there was no significant survival benefit in the NACT group compared with the pTRS group [31].

In light of the discrepancy between the European clinical trials and U.S. observational study (Table 1 and Table 2), there is an ongoing multicenter, international, randomized controlled trial at 20 study locations in Europe (Austria, Denmark, France, Germany, Italy, Sweden and United Kingdom) and United States (NCT02828618) [32]. Eligibility requires stage IIIB-IVB ovarian, fallopian tubal and primary peritoneal cancer and exposure allocation is NACT followed by iTRS versus pTRS followed by postoperative chemotherapy. Primary endpoint is OS. This is a superiority trial, testing the hypothesis that pTRS is associated with improved outcome compared to NACT-iTRS. Notably, the participating sites are restricted to centers with ≥50% complete resection rates and ≥36 TRS per year; results are expected in 2024.

The number of cycles of neoadjuvant chemotherapy prior to iTRS has also been a subject of debate. General treatment strategies for NACT include three to four cycles of platinum-based chemotherapy before iTRS; however, some patients have required up to six cycles of platinum-based NACT prior to iTRS depending on the burden of disease and response to therapy. Some studies showed that the effect of six cycles of NACT+ iTRS on survival was equivalent to the effect of three cycles of NACT+ iTRS+ three cycles of postoperative chemotherapy among patients with advanced ovarian cancers [33]. However, other studies have reported that more than three cycles of NACT before iTRS were associated with worse overall survival [34]. Since the role of NACT is still debatable, it is important to identify patients who will derive the most benefit from NACT followed by iTRS.

## 3. Potential Disadvantages of NACT

Along with the benefits mentioned above, NACT has limitations including potential emergence of chemoresistance and high recurrence rates. Some studies have reported that patients treated with NACT followed by iTRS had higher rates of platinum resistance and relapse than patients treated with pTRS [35,36,37]. The reason behind the development of resistance could be higher disease burden at the time chemotherapy was started. Due to poor blood supply, large tumors may not get adequate chemotherapy and develop resistance and new mutations [35]. Treatment interruption in the middle of chemotherapy for iTRS could also affect drug resistance [25,35]. Unrecognized residual cancer cells during iTRS, increased amounts of ovarian cancer stem cells and NACT-induced gene mutations might be mechanisms for platinum resistance in patients treated with NACT [37]. Elevated levels of aldehyde dehydrogenase 1 (ALDH1)-positive cancer stem cells after NACT may be associated with early relapse (within 6 months of treatment completion) and platinum resistance [38]. In an analysis of in vitro drug resistance assays, the tumors of women who received NACT were more likely to develop extreme resistant to platinum compared to tumors of those who did not (cisplatin extreme resistance 30% vs. 7.3%, *p* = 0.027; and carboplatin extreme resistance 33.3% vs. 9.2%, *p* = 0.038) [39]. However, some studies have found no relationship between first-line NACT and increased platinum resistance [28].

The generally accepted time interval from last platinum-based chemotherapy to iTRS is 3–4 weeks (with neutrophils within normal rage); the range of time interval is 3–8 weeks. Some studies show that more than 4 weeks of delay in iTRS after NACT due to chemotherapy related toxicities was associated with poor PFS among patients with advanced ovarian cancer [40]. The median time interval from last platinum-based chemotherapy to iTRS was 34 days and the mean was 36.9 days. More than 6 weeks of time interval from last platinum-based chemotherapy to iTRS was defined as delay in surgery. Delay in iTRS was associated with poor OS in univariate analysis; however, delay in iTRS was not associated with poor OS in multivariate analysis after adjusting for FIGO stage, complete resection and age of patients with advanced ovarian cancer. Logistical limits, financial clearance, extended recovery from chemotherapy, comorbidities and patient’s choice were associated factors for delay in iTRS [41].

## 4. Optimal Cytoreduction

Optimal cytoreduction is important for improving survival of patients with epithelial ovarian cancer. Satisfactory cytoreductive surgery aims for no visible residual disease or residual tumor lesions of <1 cm in diameter after cytoreductive surgery [42]. Many prospective and retrospective studies have demonstrated that complete surgery/removal of all macroscopic disease has the greatest impact on patients’ survival; thus, optimal cytoreduction became the main goal of surgery. Patients with suboptimal cytoreduction with any macroscopic (>1 cm) RD had worse prognosis than those who had optimal cytoreduction (no gross residual or <1 cm) [43,44,45].

Despite the fact that most of the patients treated with NACT were diagnosed with advanced-stage disease, 60.6% of patients achieved optimal cytoreduction at iTRS following NACT vs. 38.7% of patients in the pTRS group [46]. Four randomized trials comparing NACT followed by iTRS and pTRS demonstrated higher optimal cytoreduction rates in the NACT group than in the pTRS group (in the EORTC-55971 trial, approximately 81% vs. 42%; in the CHORUS trial, 73% vs. 41%; in the Japan Clinical Oncology Group study (JCOG-0602) trial, 82% vs. 37%; and in the SCORPION trial, 58% vs. 46%, respectively) (Figure 1) [17,18,21,22]. Complete cytoreduction after iTRS was associated with the highest median OS (58 months) and PFS (14 months). Patients who had suboptimal cytoreduction after iTRS had the lowest median OS (33 months) and PFS (6 months) [47]. The definition of surgical outcome (amount of residual disease after surgery) is subjective and could be affected by surgeon’s skills, organization’s infrastructure and ability to identify suitable patients [48].

## 5. Potential Models to Guide Upfront Decision-Making

Several studies have been conducted to assess various prognostic models based on clinical factors, radiology studies, laparoscopic triage, circulating molecular markers and tumor-based genetic markers. Here, we summarize proposed investigative models that can direct treatment decision-making for patients with suspected ovarian cancer in an upfront setting. We have summarized important decision-making criteria for NACT in Table 3.

### 5.1. Clinical Factors

#### 5.1.1. Models Based on Various Clinical Factors

All patients with stage IIIC or IV ovarian cancer should be evaluated by a gynecologic oncologist for selection of the most appropriate primary treatment. The European Society for Medical Oncology in 2013 recommended NACT for patients with poor performance status, extensive tumor dissemination and low albumin levels [49]. Some studies have suggested that pathologically confirmed (cytological examination or biopsy) malignant tumor with pleural effusion, cancers with extensive intraperitoneal metastases, patients with comorbidities who were not medically fit for primary surgery and an absence of acute intestinal obstruction and other symptoms of emergency surgery were probable indications for NACT [43,46].

Furthermore, Wright and colleagues recommended that patients with a low probability of optimal cytoreduction or with a high perioperative morbidity risk should be offered NACT. Risk factors for perioperative morbidity or mortality included advanced age, poor performance status, higher body mass index, poor nutritional status, low albumin level, ascites, multiple comorbidities, recent venous thromboembolism and advanced cancer stage (such as unresectable parenchymal liver metastasis, metastasis to the lungs or mediastinum, mesenteric retraction, bulky periportal lymph nodes or unresectable extra abdominal lymph nodes) [50].

Study findings have been mixed on the selection of treatment for stage III ovarian cancer. Exploratory analyses of the EORTC-55971 trial found that clinical staging is associated with benefits from NACT. Patients with stage IV cancer and large metastatic tumors (>45 mm) had better 5-year survival rates (23%) with NACT than with pTRS (2%). Patients with stage III cancer with metastatic tumors (>45 mm) or with stage IV cancer with metastatic tumors (≤45 mm) showed no significant difference between the two treatments [51]. Patients with stage IIIC cancer and the largest metastatic tumor of <5 cm showed better PFS with pTRS than with NACT, but no differences were found for OS between these groups [19]. Similar association for stage III and IV diseases were observed in a U.S. observational study as above [11].

A higher amount of ascites at the time of primary diagnosis was related to lower chances of optimal cytoreduction. Specifically, ascites ≥1000 mL was related to lower OS and a lower rate of optimal cytoreduction among patients with HGSOC. The presence of ≤200 mL of ascites was related to longer OS and improved surgical outcome [52]. Ascites regression (residual ascites <500 mL) in patients with HGSOC indicated better OS and PFS and a higher optimal iTRS cytoreduction rate [53]. Tumor factors to consider before the use of NACT include histology grade, tumor stage, cytology of ascites, the presence/absence of viable cancer cells and probability of optimal cytoreduction [54]. Other factors such as advanced age, race, a Charlson–Deyo Comorbidity Index of 1, performance status, frailty, socioeconomic status, distance of patients’ residence from the treating hospital and treatment at academic medical institutes or comprehensive community cancer centers were related to the use of NACT [55].

Tumor histologic confirmation is an important factor to differentiate between low-grade and high-grade tumors before primary treatment since low-grade tumors have been proven to be less sensitive to chemotherapy than high-grade tumors [47]. Another purpose of the histologic confirmation prior to NACT initiation is to rule out other cancer origin (e.g., gastro-intestinal). Other studies have demonstrated that serous types of ovarian cancers are more chemosensitive and show better response to platinum-based chemotherapy than clear cell or mucinous types [56,57]. As advanced clear cell/mucinous ovarian cancer has distinct survival compared to advanced HGSOC, careful assessment and counseling is warranted when NACT is considered [58].

#### 5.1.2. Models Based on Radiology Studies

Preoperative imaging studies are commonly used to evaluate tumor burden and to predict optimal/suboptimal cytoreduction. Imaging methods are also an important diagnostic tool in determining cancer prognosis and the effectiveness of therapy in cancer patients.

Computed tomography (CT) is the most common imaging technique used for tumor assessments because of its widespread coverage and fast scanning [59]. Inability to detect small-size peritoneal, mesenteric and visceral lesions; lack of accuracy in classifying lymph nodes; and poorer soft tissue contrast are identified limitations of CT imaging [59]. Borley and associates [60] conducted a two-phase retrospective study; presence of disease sites such as pleural effusion, lung metastasis, small-bowel mesentery deposits, large-bowel mesentery deposits and metastasis to inferior renal para-aortic lymph nodes, on preoperative CT, were significantly associated with suboptimal cytoreduction in patients with advanced-stage ovarian cancer (sensitivity of 69.2% and specificity of 71.4%). They also concluded that multiple deposits of >10 mm on bowel mesentery could be associated with a higher chance of suboptimal resection and of multiple bowel resections [60]. Some retrospective studies suggested that variables such as diffuse peritoneal thickening [61], large-volume ascites noted on preoperative CT [61], metastasis to inguinal or pelvic lymph nodes [62] and omental extension to the stomach or spleen [62] decreased the chances of optimal cytoreduction.

A prospective multicenter trial of preoperative CT and cancer antigen 125 (CA-125) developed a model (including nine criteria; Table 4) for predicting suboptimal cytoreduction (RD > 1 cm) [63]. The overall predictive accuracy of this model was 75.8%. Based on predictive scores of 0, 1–2, 3–4, 5–6, 7–8 and 9 and higher, the suboptimal cytoreduction rates were 5%, 10%, 17%, 34%, 52% and 74%, respectively [63]. A subsequent model incorporating 11 criteria (Table 4) was explored for its usefulness in predicting gross RD and in planning personalized treatment based on analysis of collected data [64]. The overall accuracy of this model was 72%. Patients who had higher predictive scores had higher rates of RD after tumor reductive surgery. The gross RD rates were 45%, 68%, 87% and 96% relative to the predictive sores of 0–2, 3–5, 6–8 and ≥9, respectively. These results suggested that both models could be integrated to determine treatment option for patients with ovarian cancer, depending on the possibility of achieving optimal or suboptimal cytoreduction [64]. Kumar and colleagues [65] conducted a validation study for both of the above-mentioned predictive models (a model to predict RD > 1 cm and a model to predict gross RD). They were able to validate the CT predictive model (with 11 criteria) to estimate gross RD at their center, but they could not validate the first model to estimate RD > 1 cm.

A clinical study used the CT evaluation model with serum CA-125 level to predict the primary treatment for patients with advanced epithelial ovarian cancer. Quin and colleagues [66] established a new scoring system (detailed scoring parameters are described in Table 4) based on previous models (Bristow’s study and Vorgias’ study [61]). There were no significant differences in optimal tumor cytoreduction, postoperative complication rate, operative time or intraoperative blood loss among patients with cumulative scores of <5. The optimal cytoreduction rate was higher and the operative time, intraoperative blood loss and postoperative complication rate were significantly lower among patients treated with NACT followed by iTRS with predictive scores of ≥5 [66].

A retrospective study [67] compared the accuracy of CT and magnetic resonance imaging (MRI) techniques in identifying preoperative inoperable cancer sites (Table 4). These imaging techniques showed sensitivity of 76%, specificity of 99%, positive predictive value of 94% and negative predictive value of 96% for estimation of suboptimal cytoreduction. CT and MRI were equally effective (*p* = 1.0) in the detection of inoperable tumors; thus, both techniques are helpful in selecting pTRS vs. NACT as the primary treatment for newly diagnosed ovarian cancer [67].

Another study [68] compared CT and MRI techniques in predicting the peritoneal cancer index (PCI; a numeric score that assesses the extent of intraperitoneal disease in 13 regions of the abdomen and pelvis, with a maximum score of 39) among three groups (small-volume tumors: PCI score 0–9; moderate-volume tumors: PCI score 10–20; and large-volume tumors: PCI score > 20) of patients with ovarian cancer. Results demonstrated that MRI identified 91% of tumors correctly, whereas CT identified 50% of tumor correctly. MRI (95% sensitivity, 70% specificity and 88% accuracy) was found to be superior to CT (55% sensitivity, 86% specificity and 63% accuracy) in the detection of individual peritoneal sites [68]. Rizzo and associates [69] revealed that whole-body diffusion-weighted MRI had significantly better accuracy than CT had in detecting tumor sites such as carcinomatosis in the mesentery, large bowel, sigmoid colon, pelvis and lumboaortic and pericardiophrenic lymph nodes. Multivariate analysis showed that mesenteric carcinomatosis, mesenteric retraction and carcinomatosis in the small and large bowel were statistically significant criteria for suboptimal cytoreduction [69]. The limitations of MRI include high cost, longer examination time, inadequate resolution for large field coverage and susceptibility to artifacts (breathing movements and bowel motion) [59].

Some researchers [70] have found a significant association between the estimation of operability of tumors and fluorodeoxyglucose positron emission tomography/computed tomography (FDG-PET/CT) findings of tumor sites including duodenum and pancreas, hepatic hilum and root of mesentery. Patients with higher chances of optimal cytoreduction were offered pTRS and patients with higher chances of suboptimal cytoreduction were offered NACT. FDG-PET/CT sensitivity was 91%, specificity was 67% and accuracy was 86% [70]. Chong and colleagues [71] evaluated the metabolic parameters (individual SUVmax values and the sum of the SUVmax values of nine regions of the abdomen) of F-18 FDG-PET/CT to estimate suboptimal cytoreduction in patients with advanced ovarian cancer. Results showed that the sum SUVmax of the nine regions plus the SUVmax of lymph nodes; ECOG performance status; the SUVmax of right upper, central and left upper abdominal regions; and hypermetabolic lesions in areas of small bowel mesentery, omentum, liver, diaphragm, spleen, stomach or lesser sac were related to suboptimal cytoreduction. The PFS and OS rates were worse in the higher-risk group (predictive score > 10) than in the lower-risk group (predictive score ≤ 10) [71]. FDG-PET/CT can detect exact location of cancer lesions and scan the whole body for metastasis, but FDG uptake may be affected by body’s metabolic activities and glucose transport receptors [59].

#### 5.1.3. Models Based on Laparoscopic Triage

Laparoscopic scoring models have been found to be reliable for assessing the extent of disease. The benefits of laparoscopy encompass extensive abdominopelvic visualization including the diaphragm and liver, faster recovery to initiate personalized treatment and lower complication rates. Fagotti and colleagues [72] demonstrated that the effectiveness of the diagnostic laparoscopic staging model is similar to that of standard laparotomy for predicting intraperitoneal disease dissemination and optimal cytoreduction in patients with ovarian cancer (Table 5). They developed and confirmed the laparoscopy-based quantitative predictive model in a prospective study of patients with ovarian cancer. The predictive index value (PIV) was calculated on the basis of laparoscopic parameters including stomach infiltration, diaphragmatic carcinomatosis, peritoneal carcinomatosis, omental caking, mesenteric retraction, bowel infiltration and superficial liver metastases. The presence of any parameter was scored 2 and the absence of the parameter was scored 0. Patients with a PIV of ≥8 experienced suboptimal cytoreduction with a specificity of 100%, positive predictive value of 100% and negative predictive value of 70% [72].

The accuracy and reliability of this model was further validated in a prospective study at multiple centers [74]. Mesenteric retraction was the least accurate laparoscopic variable in assessing intraperitoneal tumor spread in patients with ovarian cancer. Peritoneal carcinomatosis and bowel infiltration ranged from 99.2% to 90% accuracy. The inter-rater reliability (Cohen’s kappa) was 0.685; the P value was 0.01; and the accuracy was 84.1%, which after analysis adjustment improved the Cohen kappa to 0.773; the *P* value to 0.388; and the accuracy to 88.6%. The study concluded that the laparoscopic scoring model was accurate and reproducible for identification of intraperitoneal tumor spread and prediction of optimal cytoreduction [74]. One retrospective study [75] developed an updated laparoscopic scoring algorithm, with PIV cut-off score of 10, for predicting suboptimal cytoreduction in patients with advanced-stage ovarian cancer. Discriminating performance was increased (area under the curve = 0.885) and the rate of unnecessary laparotomies were decreased with this algorithm. The study also noted that the probability of achieving complete cytoreduction was negligible in patients with PIV score of ≥10 [75]. Later, Fagotti and colleagues [21] examined the superiority of NACT followed by iTRS over pTRS with regard to quality of life and perioperative morbidity for patients with advanced ovarian cancer. Patients with a laparoscopy PIV of ≥8 or ≤12 (considered as having a high tumor load) were included and randomized to either the pTRS or NACT group. The pTRS group had higher perioperative comorbidities; thus, NACT could be a better choice for patients with very high tumor load. There were no significant differences in terms of quality of life between the two groups [21].

We conducted a prospective quality-improvement study to evaluate the effects of the laparoscopic staging model for personalizing the selection of a surgical approach. CT imaging, laparoscopic scoring (Table 5) and blinded fashion two surgeons’ scoring were used as the criteria with which patients were triaged to either non-scope/NACT group, scope/NACT group or pTRS group [73]. Patients with medically or surgically inoperable disease were treated with NACT (the non-scope/NACT group); patients with a PIV of ≥8 were triaged to NACT (the scope/NACT group); and patients with a PIV of <8 were treated with pTRS (the pTRS group). Eighty-three percent of patients from the NACT group underwent iTRS. The laparoscopic scoring algorithm and our team’s operating efforts improved the complete cytoreduction (R0 resection) rate to 88% in the pTRS group and to 74% in the NACT group. A subgroup analysis revealed that the complete cytoreduction rate was 81% in the non-scope/NACT group and 76% in the scope/NACT group. Improved complete (R0) cytoreduction rates resulted in a median PFS of 23.5 months in the pTRS group and 15.5 months in the NACT group (*p* < 0.001). Baseline serum CA-125 and gross residual tumor were identified as independent factors for predicting PFS by multivariate analysis in patients who underwent laparoscopic scoring. There was a 2% gastrointestinal complication rate associated with laparoscopy (all were trocar entry injury). Thus, laparoscopic assessment offered more individualized treatment options in patients with advanced ovarian cancer and improved PFS in all patient groups [73].

A retrospective study evaluated the effects of the sequential approach of preoperative CT imaging, frailty assessment and diagnostic laparoscopic staging in selecting appropriate treatment for patients with advanced ovarian cancer. Patients without high tumor load were offered pTRS. High-risk patients (age > 75 years, American Society of Anesthesiologists (ASA) class ≥3, distant metastases and positive ascites cytology) were treated with NACT. Other patients were offered laparoscopic scoring assessment (the diagnostic laparoscopy scoring group) to decide on pTRS (PIV of <8) vs. NACT. The diagnostic laparoscopy scoring group had shorter median operative times, less frequent need for transfusions and fewer intensive care hospitalization. Thus, the proposed sequential approach could be helpful in selecting appropriate treatment and avoiding needless laparotomies and presumptive complications [76].

Andikyan and colleagues [77] assessed the efficacy and safety of the laparoscopic procedure in predicting optimal cytoreduction among patients with advanced ovarian cancer. The laparoscopic procedure was 98% sensitive in predicting optimal cytoreduction and was not associated with serious surgical complications. Peritoneal carcinomatosis, mesenteric involvement, omental caking and bowel infiltration were found to be important indicators for non-resectable disease. The optimal cytoreduction rate was 89% in patients who underwent tumor reductive surgery. The median OS of 49 months in study patients suggested that adding the laparoscopic procedure before tumor reductive surgery was not associated to lower survival rates [77]. A prospective study compared the accuracy of multidetector CT and laparoscopy in terms of predicting intraperitoneal carcinomatosis by calculating the PCI score. The sensitivity, specificity, accuracy, positive predictive value and negative predictive value for multidetector CT were 94.9%, 86.7%, 93.8%, 97.9% and 72.2%, respectively and for laparoscopy were 98.3%, 80.4%, 95.7%, 96.8% and 88.8%, respectively. Accuracy of multidetector CT was reduced on size-based analysis (80% accuracy in detecting lesions <1-cm) and region-based analysis (50% accuracy for pelvic region and 80% accuracy for small intestine region), whereas laparoscopy showed 95% accuracy in detecting lesions <1-cm, 93% accuracy in pelvic region and 94% accuracy in small intestine region. Multidetector CT offers better wide-ranging analysis, whereas laparoscopy offers valuable information to predict suboptimal cytoreduction. In this study, the laparoscopy procedure was not associated with any serious complications [78].

### 5.2. Molecular Markers

Candidate relevant molecular markers for NACT response in ovarian cancer are shown in Table 6.

#### 5.2.1. Models Based on Circulating Molecular Markers

Serum CA-125 is the most common tumor marker used at diagnosis and subsequent surveillance visits. A retrospective study demonstrated that patients with a serum CA-125 of ≤100 U/mL after NACT (before iTRS) had a higher likelihood of achieving optimal cytoreduction [79]. Another study, however, reported that a preoperative serum CA-125 level of ≤30 U/mL was a significant predictor of optimal cytoreduction [33]. Some studies found that a post-NACT serum CA-125 level of <35 U/mL was associated with better PFS and OS [80,81] and higher chances of optimal cytoreduction [81] and was an independent prognostic factor for sensitivity to platinum therapy [80]. A post-NACT serum CA-125 level of >100 U/mL was associated with worse PFS and OS in patients with stage III or IV ovarian cancer [56]. A post-NACT serum CA-125 level of ≤100 U/mL and a >80% decrease in post-NACT serum CA-125 level were found to be predictive factors for optimal cytoreduction. A greater than 80% drop in post-NACT serum CA-125 level was also associated with an increased chance of achieving complete cytoreduction [82]. One study found that a higher ratio of serum CA-125 to ascites leptin was suggestive of baseline chemoresistance and worsened OS in patients with HGSOC (*p* = 0.023 and area under the curve [AUC] value = 0.846). Thus, the serum CA-125 to ascites leptin ratio could anticipate clinical response before primary treatment and could be helpful in selecting type of first-line treatment for patients with HGSOC [83].

One retrospective study assessed the prognostic value of human epididymis 4 (HE4) protein marker preoperatively and at various time points during primary treatment for ovarian cancer [84]. In this study, the serum HE4 level was more valuable than serum CA-125 in estimating surgical outcome after pTRS (77.4% sensitivity and 75% specificity at an HE4 level of 353.2 pmol/L) and iTRS (92.9% sensitivity and 69% specificity at an HE4 level of 154.3 pmol/L). A greater than 70% change in the value of serum HE4 after NACT was more likely associated with optimal cytoreduction [84]. The authors revealed that a combination of serum HE4 level (226 pmol/L), serum CA-125 level (89 U/mL) and CT imaging was significant in identifying RD and in determining cancer prognosis (96% sensitivity and 92% specificity). A post-NACT serum HE4 level of 226 pmol/L was helpful in categorizing patients to low or high risk of suboptimal surgery (75% sensitivity and 85% specificity) [85].

ADLH1-positive cancer stem cells have been shown to have an important role in platinum sensitivity. Increased expression of ALDH1 after NACT treatment indicated a 4.18 times higher risk of death and increased risk of poor outcome [38].

Analyses of two publicly available microarray datasets revealed that higher levels of fatty acid–binding protein 4 (FABP4) and alcohol dehydrogenase 1B (ADH1B) in primary tumors were associated with a higher incidence of RD after pTRS among patients with HGSOC [86]. In The Cancer Genome Atlas (TCGA) dataset, 90.3% of patients with high expression of FABP4 and ADH1B (cutoffs of 3.5 for both markers) showed RD. In the Tothill dataset, 93.7% of patients with high expression of FABP4 and ADH1B (cutoffs of 5.25 for FAB4 and 4.5 for ADH1B) showed RD. Levels of FABP4 were higher in metastatic tissues and a higher omental level of FABP4 was associated with a high risk of RD [86].

One study [87] analyzed expression level of microRNAs (miR-199, miR-181, miR-30, miR-29 and let-7) at the time of initial laparoscopy and at iTRS. The authors found that the levels of miR-199a-3p, miR-199a-5p, miR-181a-5p and let-7g-5p at the time of diagnosis had a strong independent association with PFS and OS, whereas miR-199b-5p had association with only OS and let-7a-5p had association with only PFS. Higher expression levels of Smad2 phosphorylation (P-Smad2), miR-181a-5p, miR-199a-5p and miR-199a-3p were associated with decreased platinum-free interval (usually <6 months), increased chance of RD >1 after iTRS and poor survival [87]. Other authors demonstrated that combinations of serum miR-34a-5p and serum CA-125 levels were strong predictors of complete resection of disease among patients with HGSOC [88].

Examination of samples from tumor tissues and ascites revealed that insulin-like growth factor (IGF)-I in ascitic fluid was an independent predictor of objective clinical response [89]. The authors also found that although IGF-II, IGF binding proteins (IGFBPs) and pregnancy-associated plasma protein-A (PAPP-A) were increased in patients with ovarian cancers, they were not linked with objective clinical response.

One study suggested that an increased level of serum calretinin (CRT), a calcium-binding protein, at the time of initial diagnosis was correlated with a higher amount of ascites and advanced FIGO staging. An increased level of serum CRT (sCRT level cutoff = 0.35 ng/mL) was associated with higher chances of suboptimal cytoreduction. Serum CRT levels were also found to be an independent predictor for PFS and OS [90].

#### 5.2.2. Models Based on Tumor-Based Genetic Markers

Results of a multicenter retrospective study [91] revealed that germline BRCA 1 or 2 gene mutations were associated with higher occurrence of peritoneal carcinomatosis and the presence of bulky lymph nodes. The presence of a BRCA1 or 2 mutation was frequently associated with a higher laparoscopic PIV score (≥8) compared with the wild-type BRCA genotype. In this study, pTRS was linked with longer PFS (26 months) compared with PFS with NACT (18 months) in patients with HGSOC and the BRCA wild-type genotype [91]. Some researchers [92] concluded that the chemotherapy response score could be used to estimate survival in patients with BRCA wild-type ovarian cancer. Patients with wild-type BRCA with complete response had better PFS and OS. One meta-analysis [93] showed that the presence of both BRCA1 and BRCA2 mutations was related to increased complete response rate, improved OS and decreased partial response rate with ovarian cancer.

In another study [94], quantitative analysis of study samples showed that patients with c-myc expression of >200 had a better 5-year survival rate. Multivariate analysis of samples showed that c-myc expression was an independent prognostic factor.

## 6. Conclusions

The heterogeneity and later stage of diagnosis make curative treatment difficult for patients with ovarian cancer. NACT followed by iTRS has been shown to be as effective as pTRS for advanced-stage ovarian cancers. NACT can be considered a primary treatment option for patients with FIGO stage IV and is preferred for patients with FIGO stage IIIc ovarian cancer for whom optimal cytoreduction is not achievable or for those with multiple comorbidities. Many models have been proposed based on clinical factors, radiology studies, laparoscopic triage, circulating molecular markers and tumor-based genetic markers to facilitate upfront decision-making. Among these, laparoscopic assessment is arguably the most informative surgical assessment tool available. Additional research is required to determine more reliable models and to direct decision-making in upfront clinical management for advanced epithelial ovarian cancer.

## Figures and Tables

**Table 1:** Summary of RCTs comparing NACT to pTRS in advanced epithelial ovarian cancer.

Author	[20]		[21] ^#^		[17]		[18]	
Year	2020		2016		2015		2010	
Enrolled cases	NACT	pTRS	NACT	pTRS	NACT	pTRS	NACT	pTRS
	149	152	55	55	274	276	334	336
Stage IV	49 (32.9)	47 (30.9)	4 (7.3)	8 (14.5)	68 (24.8)	70 (25.4)	81 (24.3)	77 (22.9)
PS 0–1	131 (86.2)	130 (87.2)	50 (90.9)	51 (92.7)	221 (80.7)	221 (80.1)	290 (86.8)	294 (87.5)
PS ≥ 2	21 (13.8)	19 (12.8)	5 (9.1)	4 (7.3)	53 (19.3)	54 (19.6)	44 (13.2)	40 (11.9)
Surgical time (mins)	302	240	275	451	120	120	180	165
R0 ^‡^	83 (63.8)	17 (11.6)	30 (57.7)	25 (45.5)	79 (39.3)	39 (16.7)	151 (51.2)	61 (19.4)
Periop mortality	0	1 (0.7)	0	2 (3.6)	1 (0.5)	14 (5.5)	2 (0.7)	8 (2.5)
G3-4 AE	7 (5.4)	25 (17.0)	3 (5.8)	27 (49.1)	30 (14)	60 (24)	17 (5.3) *	56 (18.1) *
DFS	HR 0.96 (0.75–1.23)		HR 1.06 (0.77–1.46) ^†^		HR 0.91 (0.76–1.09)		HR 1.01 (0.89–1.15) ^¶^	
OS	HR 1.05 (0.84–1.33) ^§^		-	-	HR 0.87 (0.72–1.05)		HR 0.98 (0.84–1.13) ^¶^	
**Subgroup analysis of overall survival in the NACT group as compared with the pTRS group**								
Age > 70	-	-	-	-	Comparable	-	Comparable	-
PS ≥ 2	Comparable	-	-	-	Comparable	-	Comparable	-
Stage IIIC	Comparable	-	-	-	Comparable	-	Comparable	-
Stage IV	Comparable	-	-	-	Comparable	-	NACT better	-
CSS	-	-	-	-	-	-	-	-
Other cause of death	-	-	-	-	-	-	-	-
Non-serous ^$^	Comparable	-	-	-	-	-	Comparable	-
R0	-	-	-	-	Comparable	-	Comparable	-

Adopted and modified from the author’s own work [11]; *Gynecol. Oncol.* **2021**, *160*, 32–39. Matsuo, K., et al. Possible candidate population for neoadjuvant chemotherapy in women with advanced ovarian cancer. Copyright (2021) with permission from Elsevier. Summary of randomized control trials comparing NACT to pTRS in advanced epithelial ovarian cancer. Number (percentage per column) of hazard ratio (95%CI) is shown. ^#^ women with predictive index >8 or <12 were included. * included hemorrhage, infection and venous complication. ^†^ this result was reported in 2018. ^‡^ no macroscopic residual disease. ^$^ indicates mucinous carcinoma and clear cell carcinoma. ^§^ this study could not confirm the noninferior OS of NACT. ^¶^ 90% confidence interval. The 95%CI overlaps 1 and the upper bound of the 95% CI exceeds the predetermined non-inferior margin (1.161). Abbreviations: RCT, randomized controlled trial; NACT, neoadjuvant chemotherapy; pTRS, primary cytoreductive surgery; Op, operation; G3 or G4, grade 3 or grade 4; AE, adverse event; DFS, disease free survival; OS, overall survival; CSS, cause specific survival; -, not applicable; HR, hazard ratio; and CI, confidence interval.

**Table 2:** Summary of population-based studies comparing NACT to pTRS in advanced epithelial ovarian cancer.

Author	[11]	[7]	[29]	[9]	[30]	[6]	[10]	[8]
Year	2021	2020	2020	2018	2017	2016	2016	2014
Period	2010–2016	2004–2016	2004–2015	2000–2013	2003–2011	2004–2013	2003–2012	1991–2007
Data base	SEER	NCDB	NCDB	SEER-Medicare	NCDB	NCDB	NCCN **	SEER-Medicare
No.	4360	72171	36602	5417	22962	40694	1538	9587
NACT	1268 (29.1)	19150 (26.5)	9885 (27.0)	1221 (22.5)	3126 (13.6)	5429 (13.3)	416 (27.0)	2238 (23.3)
Age	Any	Any	Any	≥66	≤70	≥40	Any	≥65
CCI	Any	Any	Any	Any	0	Any	Any	Any
Stage	III, IV	IIIC, IV	III, IV	III, IV	IIIC, IV	IIIC, IV	IIIC, IV	II-IV
R0 ^†^	-	-	65.4 vs. 56.1 ^#^	-	-	-	36.8 vs. 20.8	-
Use of NACT (%)	29.7 in2010 38.9 in 2016	17.6 in 2004 * 45.1 in 2016 *	-	16 in 2000 35.4 in 2013	-	8.6 in 2004 22.6 in 2013	27 in 2003 * 49 in 2012 *	19.7 in 1991 * 31.8 in 2007 *
*p*-trend	*p* < 0.001	*p* < 0.001 ^	-	*p* < 0.0001	-	*p* < 0.001	*p* < 0.01	*p* < 0.0001
OS ^¶^	pTRS better	-	pTRS better	-	pTRS better	-	-	pTRS better
**Subgroup analysis of overall survival in the NACT group as compared with the pTRS group**								
Age > 70	Comparable	-	-	pTRS better ^‡^	-	-	-	-
Stage IIIC	pTRS better ^&^	-	-	pTRS better	pTRS better	-	pTRS better	-
Stage IV	Comparable	-	-	Comparable	pTRS better	-	Comparable	-
Serous	-	-	-	-	pTRS better	-	-	-
HVC	-	-	-	-	pTRS better	-	-	-
R0	-	-	pTRS better	-	-	-	Comparable	-

Adopted and modified from author’s own work [11]: *Gynecol. Oncol.* **2021**, *160*, 32–39. Matsuo, K., et al. Possible candidate population for neoadjuvant chemotherapy in women with advanced ovarian cancer. Copyright (2021) with permission from Elsevier. Summary of population-based studies comparing NACT to pTRS in advanced epithelial ovarian cancer. Number (percentage per column) is shown. Salient observational studies in the United States are examined. * In the trend analysis, neoadjuvant chemotherapy without surgery was included. ** The National Comprehensive Cancer Center Network (NCCN) Ovarian Cancer Outcomes Database. ^&^ Stage III. ^†^ indicates no residual disease. ^#^ microscopic or no residual disease. ^‡^ Comparable at age ≥ 80 years. ^¶^ Stage III and IV (pTRS versus NACT + interval cytoreductive surgery). ^ *p*-value for trend of change between 2011 to 2016 was 0.01. Abbreviations: NACT, neoadjuvant chemotherapy; pTRS, primary cytoreductive surgery; CCI, Charlson Comorbidity Index; OS, overall survival; NCDB, National Cancer Database; SEER, The Surveillance, Epidemiology and End Results, Serous, serous histology; -, not applicable; and HVC, high volume center.

**Table 3:** Decision-making criteria for use of NACT.

Biopsy-confirmed FIGO stage IV advanced epithelial ovarian, fallopian tube and peritoneal cancers Biopsy-confirmed FIGO stage IIIC advanced epithelial ovarian, fallopian tube and peritoneal cancers who are not fit for surgery
High-grade serous type of advanced epithelial ovarian, fallopian tube and peritoneal cancers
Higher perioperative morbidity or mortality: Poor performance status, advanced age, higher body mass index, poor nutritional status, low albumin, high-volume ascites, multiple comorbidities
Extensive intraperitoneal or extraperitoneal metastases such as large metastatic tumors (>45 mm), nonresectable parenchymal liver metastasis, metastasis to the lungs or mediastinum, mesenteric retraction, bulky periportal lymph nodes or unresectable extra abdominal lymph nodes, pleural effusion
Absence of acute intestinal obstruction or other symptoms of emergency surgery
Low possibility of optimal cytoreduction (<1 cm of residual disease)
CT findings: >2 cm diaphragm or lung base disease or confluent disease; Presence of ascites on most (2/3) of the CT scan cuts; Any size of liver parenchymal lesion or ≥2 cm surface liver lesion; ≥2 cm small or large bowel mesentery disease; Involvement of the porta hepatis or ≥1 cm disease in gallbladder fossa; Diffuse peritoneal thickening or ≥2 cm peritoneal lesions; ≥1 cm suprarenal or ≥2 cm infrarenal paraaortic lymph nodes; ≥2 cm inguinal canal disease or lymph nodes
Social factors: Distance of patients’ residence from the treating hospital, academic medical institutes or comprehensive community cancer centers.

**Table 4:** Detailed criteria of models based on radiology studies.

Author (Year)	Criteria
[63] (2014)	9 criteria: 3 clinical criteria (age ≥ 60 years, CA-125 ≥ 500 U/mL, American Society of Anesthesiologists [ASA] class ≥3) and 6 radiologic criteria (>1 cm lesions in the small bowel mesentery; >1 cm lesions in the root of the superior mesenteric artery; >1 cm lesions in the perisplenic area; >1 cm lesions in the lesser sac; >1 cm suprarenal retroperitoneal lymph nodes; and diffuse small bowel adhesions/thickening).
[64] (2017)	11 criteria: 3 clinical criteria (age ≥ 60 years, CA-125 ≥ 600 U/mL, American Society of Anesthesiologists (ASA) class-≥3) and 8 radiologic criteria (>1 cm lesions in the root of the superior mesenteric artery; >1 cm lesions in the splenic hilum/ligaments; >1 cm retroperitoneal lymph nodes above the renal hilum including supradiaphragmatic lymph nodes; >1 cm lesser sac lesions; diffuse small bowel adhesions/thickening; moderate-severe abdominal ascites; lesions on gastrohepatic ligament/porta hepatis; and gallbladder fossa/intersegmental fissure lesions).
[66] (2018)	Scoring parameters: CA-125 level (≥500 U/mL); performance status of ≥2; large-volume ascites; omentum disease extension to the stomach, spleen or lesser sac; tumor extension to the pelvic sidewall, parametria or hydroureter; peritoneal thickening; ≥2 cm peritoneal implants; ≥1 cm suprarenal paraaortic lymph nodes; ≥2 cm diaphragm or lung base disease or confluent plaques; ≥2 cm inguinal canal disease or lymph nodes; ≥2 cm liver lesion on the surface or any size parenchymal lesion; porta hepatis or ≥1 cm gallbladder fossa disease; ≥2 cm infrarenal paraaortic lymph nodes; and ≥2 cm small or large bowel mesentery disease
[67] (2005)	Preoperative inoperable cancer sites: >2 cm of peritoneal implants in lesser sac, gall bladder fossa, gastrosplenic ligament, gastrohepatic ligament, root of the small bowel mesentery, subphrenic space, intersegmental fissure or porta hepatis; >2 cm of retroperitoneal adenopathy above the renal hilum; abdominal wall incursion; or hepatic metastases

**Table 5:** Fagotti’s laparoscopy scoring algorithm to predict optimal cytoreduction.

Cancer Parameter	Score
Stomach infiltration (obvious cancer dissemination into gastric wall)	Absent = 0 Present = 2
Diaphragmatic carcinomatosis (confluent nodules and/or extensive infiltration to diaphragmatic surface)	Absent = 0 Present = 2
Mesenteric retraction (involvement of the root of the mesentery and/or large infiltrating nodules)	Absent = 0 Present = 2
Omental cake (tumor dissemination of omentum to the small and large curvatures of the stomach)	Absent = 0 Present = 2
Peritoneal carcinomatosis (enormous peritoneal diffusion and/or disease spread with miliary distribution pattern)	Absent = 0 Present = 2
Bowel infiltration (tumor dissemination to small or large bowel necessitating colon resection (except rectosigmoid colon)	Absent = 0 Present = 2
Liver metastases (superficial lesions >2 cm)	Absent = 0 Present = 2

The results are generated based on prior studies [72,73].

**Table 6:** Salient molecular markers for NACT response prediction.

Biomarker	Summary
CA-125	Serum CA-125 is the most common tumor marker used at diagnosis and to observe treatment response. A >80% decrease in serum CA-125 level after NACT is found to be associated with optimal cytoreduction. Cut-off level to measure response/progression is still debatable.
Leptin	Higher serum CA-125 to ascites leptin ratio is found to be suggestive of baseline chemoresistance.
HE4	Serum HE4 level is found to be more valuable tumor marker in estimating surgery outcome. A >70% decrease in serum HE4 level after NACT is found to be associated with optimal cytoreduction.
ADLH1	Higher ALDH1 level after NACT is found to be associated with poor outcome and higher risk of death.
ADH1B	Higher preoperative ADH1B level is found be associated with higher chances of RD after tumor reductive surgery.
FABP4	Higher preoperative FABP4 level is found be associated with higher chances of RD after tumor reductive surgery.
MicroRNA	Higher level of specific MicroRNAs (Smad2 phosphorylation (P-Smad2), miR-181a-5p, miR-199a-5p and miR-199a-3p) is found to be associated with higher chances of RD after iTRS, decreased platinum-free interval and poor survival.
IGF-I	The presence of IGF-I in ascitic fluid is found to be an independent predictor of objective clinical response.
Calretinin	Higher serum CRT level is found to be associated with higher chances of suboptimal cytoreduction.
BRCA1/2	The presence of BRCA1/2 is found to be associated with higher chances of optimal cytoreduction and better survival.
c-Myc	c-Myc expression of >200 is found to be associated with better 5-year survival rate.
